# Early detection of postoperative infections using continuous temperature monitoring: A prospective clinical trial

**DOI:** 10.1007/s10877-025-01383-y

**Published:** 2025-12-05

**Authors:** Lars Schäfer, Franziska Dickel, Karl Strohmayer, Werner Koele, Bettina Leber, Robert Sucher, Philipp Stiegler

**Affiliations:** 1 Department of Surgery, Medical University of Graz, Graz, Styria Austria; 2 SteadySense GmbH, Seiersberg-Pirka, Styria, Austria

**Keywords:** Continuous temperature monitoring, Wearable patch, Postoperative infection, Digital health, Nosocomial infection detection, Patient monitoring technology

## Abstract

This study aimed to evaluate whether continuous axillary temperature monitoring using a wearable patch enables earlier detection of postoperative infections compared to conventional intermittent infrared thermometry. 103 surgical patients were included in this prospective, single-center study and monitored over an 11-month period. Continuous axillary temperature monitoring using the SteadyTemp^®^ patch was compared to routine infrared measurements performed as part of clinical routine. The primary outcome was fever detection rate (≥ 38.0 °C). Secondary outcomes included the correlation between fever detection and laboratory values as well as the frequency of clinical interventions. Out of 103 included patients, fever was detected in 33 cases. Continuous monitoring identified fever in 31 of these 33 patients (93.9%), whereas infrared thermometry detected fever in only 12 cases (36.4%). In 16 cases where antibiotic therapy was initiated or adjusted due to newly detected fever, the patch detected fever in 15 patients, compared to only 7 detections by infrared thermometry. Surgical interventions due to suspected infections were performed in 5 patients, and fever was detected by the patch in all cases, while infrared thermometry detected fever in only 2 of these patients. Due to the frequent failure of infrared thermometry to detect fever, a scoring system was developed to assess the clinical relevance of fever detection. Continuous temperature monitoring with the SteadyTemp^®^ patch demonstrated superior fever detection compared to infrared thermometry, leading to earlier identification of febrile events. This study suggests that continuous temperature monitoring may enhance infection surveillance in surgical patients, allowing for more timely clinical interventions.

## Introduction

Healthcare-associated infections pose a significant burden on healthcare systems worldwide and represent a serious threat to patient safety [1, 2]. Hospitalized patients, especially those undergoing invasive procedures are at an elevated risk of infection [3]. Recent studies indicate that about 15% of ICU patients in Europe develop healthcare-associated infections, with even higher prevalence rates being reported in some developing countries [4, 5]. In Europe alone, an estimated 3.5 million patients are affected annually, leading to approximately 90,000 deaths [4]. Health-care associated infections lead to prolonged hospital stays, increased morbidity and mortality rates, and significantly higher treatment costs [1].

The most common types of nosocomial infections include surgical site infections and pneumonia [6]. The onset of these infections is associated with various factors, including immunosuppression, the use of invasive procedures and intensive care unit admission [3]. Management is further complicated by the risks associated with multidrug-resistant organisms, which account for a relevant proportion of cases [7]. Addressing these challenges often requires specialized therapeutic approaches, such as targeted antibiotic regimens and patient isolation [8].

In clinical practice, the prevention of healthcare-associated infections needs to be addressed through stringent hygiene protocols and surveillance programs [9]. Although studies show that consistent hygiene measures and prevention strategies substantially reduce infection risks, a significant residual risk remains [10]. A central approach to early detection of postoperative infections is body temperature monitoring, as fever frequently presents as one of the earliest signs of systemic infection [11]. However, traditional temperature measurement is typically conducted intermittently, potentially overlooking subtle temperature fluctuations that might indicate an emerging infection [12]. Continuous temperature monitoring using wearable sensors offers a promising solution to this issue. These technologies allow for real-time temperature tracking and could potentially deliver faster detection of early signs of infection [13].

The objective of this study was to assess the efficacy of continuous temperature monitoring via a wearable patch for the early detection of postoperative infections. We compared continuous temperature monitoring with conventional intermittent infrared thermometer measurements to assess its ability to detect infections more quickly. The goal was to assess whether fever was detected earlier, allowing for more timely intervention. Insights from this study may play a pivotal role in improving patient safety and reducing the clinical burden of nosocomial infections.

## Materials and methods

### Study design and participant criteria

This prospective, single-center observational study was approved by the local ethics committee. All participants underwent visceral surgery and were admitted to either the Interdisciplinary Surgical Intensive Care Unit (ICU) or a general surgical ward at the University Hospital of Surgery at LKH Graz. The study timeline for each participant began upon admission to the ICU or general ward and ended upon discharge.

Eligible participants included male, female, and non-binary adults who had undergone visceral surgery, were admitted to the ICU or a general ward during the study period, and were expected to have a hospital stay of at least five days. Exclusion criteria included patients under the age of 18, those with impaired decision-making capacity, individuals with a history of substance abuse, and those with known allergies to adhesive materials or patch components. Patients with chronic inflammatory conditions in their personal history, a Body Mass Index (BMI) above 33, surgical wounds within 3 cm of the planned patch site, or non-intact skin were also excluded. Chronic inflammatory conditions were predefined and excluded to reduce confounding from baseline inflammation and non-infectious fever responses and to limit effects of immunomodulatory therapy. Excluded diagnoses included rheumatoid arthritis, inflammatory bowel disease, systemic lupus erythematosus, vasculitides, autoinflammatory syndromes, and other active systemic inflammatory diseases documented in the medical record.

### Data access and blinding

Treating teams had routine access to intermittent infrared temperature measurements as part of standard care. Patch data were read out via NFC every 24–72 h and were not available in real time and therefore they were not prospectively integrated into clinical decision-making. No formal blinding was implemented, but the lack of real-time patch access minimized potential influence on management.

### Primary and secondary outcomes

The primary outcomes included the temperature charts (both continuous patch measurements and routine infrared measurements) and physician-diagnosed infections of any type.

The secondary outcomes included recorded medication use (antibiotics, anti-inflammatory drugs, antipyretics) and inflammatory markers in blood samples, specifically C-reactive protein (CRP), Procalcitonin (PCT) and leukocyte count. Blood sampling typically occurred every two days, though the frequency varied based on individual patient needs.

### Study endpoints

The primary endpoint was initially defined as the time difference (in minutes) between the first fever spike (body temperature ≥ 38 °C) detected by the continuously monitoring patch and the first spike observed through routine clinical measurements using an infrared thermometer. This definition was later revised to a point-based evaluation system designed to compare and assess the clinical relevance of fever events detected by the patch versus the infrared thermometer.

The secondary endpoint evaluated the correlation between inflammatory markers (CRP, leukocyte count, PCT) and temperature patterns recorded by the continuously monitoring patch.

### Temperature monitoring protocol

Continuous temperature monitoring was conducted using a wearable patch sensor (Steadytemp^®^). The SteadyTemp^®^ patch is an adhesive axillary thermometer which uses integrated temperature sensors to detect subtle changes in axillary skin temperature [13]. Data from the patch is read via “Near Field Communication” (NFC) every 24–72 h using a smartphone application (SteadyTemp^®^ App), allowing for monitoring and storage of temperature trends (Fig. 5).

The patch was applied to the patient’s thorax in the middle axillary line, to achieve optimal measurement quality.

Prior to placement, the patch site was cleaned and disinfected. The sensor was applied only to intact skin under one arm. The body side to which the patch was applied, as well as the number of patch replacements were documented.

In addition to this continuous monitoring, routine temperature measurements using an infrared thermometer (Model: Exergen TAT-5000 S-RS232-QR) at the temporal artery were performed daily as part of standard clinical care by nursing staff. Measurements took place at least once each day in the morning during rounds and were documented in the temperature chart. Additional measurements were performed if deemed necessary by medical staff due to clinical symptoms reported by the patient or medical indications, such as elevated inflammatory markers.

The temperature-measurement protocol on ICU was identical to that on the general ward. Because very few patients (*n* = 7) were managed on the ICU and the measurement protocol was identical to the general ward, no subgroup comparison by setting was performed; analyses were pooled across settings.

### Feasibility of statistical analysis for the primary endpoint

Due to the high frequency of undetected fever events by the infrared thermometer, a traditional statistical comparison for the primary endpoint (time difference in minutes between fever detection by the patch and the infrared thermometer) was deemed inappropriate. Instead, a points-based evaluation system was implemented to assess the clinical relevance of fever detection and thereby compare the different measurement methods clinical impact. Therefore, we developed a customized scoring system. Several established scoring systems exist in clinical medicine to evaluate patient conditions, risk stratification, and severity of illness, including the Sequential Organ Failure Assessment (SOFA) score, the National Early Warning Score (NEWS), and the Acute Physiology and Chronic Health Evaluation (APACHE) II score. These systems use weighted criteria to reflect the clinical impact of various physiological parameters. Similarly, our scoring system was designed to quantify the clinical relevance of fever detection, taking into account:

1. Duration of fever episodes (short fever spike vs. prolonged fever episode).
2. Need for antibiotic interventions (adjustments or initiation of therapy).
3. Requirement for surgical intervention.

Our approach aligns with other medical scoring systems, which assign different weights to clinical variables based on their impact on patient outcomes. For instance:

- The NEWS score assigns higher points to more extreme vital signs to prioritize early intervention.
- The SOFA score weighs organ dysfunction differently based on its severity.
- Our scoring system assigns greater weight to fever detection when it leads to a change in clinical management, reflecting its direct impact on patient care.

Unlike traditional binary fever detection models, which classify patients simply as “febrile” or “afebrile,” our system considers the clinical consequences of fever detection. By integrating intervention-based weighting, we ensure that fever episodes with higher clinical relevance are more significantly represented in the overall evaluation.

### Points-based evaluation system

- Detection of a fever spike lasting less than 60 min was assigned 1 point.
- Detection of a fever episode lasting 60 min or longer was assigned 3 points.
- Fever detection leading to an antibiotic intervention was assigned 20 extra points.
- Fever detection leading to a surgical intervention was assigned 50 extra points.

The intervention score enumerates fever-triggered events only. Interventions without documented fever, if present, would be reported descriptively and excluded from the score.

This heuristic system aimed to enable a meaningful comparison between continuous monitoring with the patch and intermittent measurements with the infrared thermometer. External validation of this system is warranted.

### Inflammatory-marker analysis (post hoc, exploratory)

We evaluated the temporal association between inflammatory markers and temperature patterns recorded by the wearable patch. Each analysis was anchored on the timestamp of the laboratory draw. For every blood draw we determined whether a febrile episode, defined as patch temperature ≥ 38.0 °C sustained for ≥ 60 min, had occurred in the preceding 36 h and whether the corresponding marker exceeded a clinically relevant threshold (CRP > 100 mg/L, PCT > 0.5 ng/mL, leukocytes < 4 × 10^9/L or > 12 × 10^9/L). Blood draws within the first 12 h after initiation of patch recording were excluded. Thresholds were chosen to align with bedside practice and widely used clinical criteria. CRP > 100 mg/L is commonly associated with clinically meaningful bacterial inflammation beyond routine postoperative response. PCT > 0.5 ng/mL is a commonly adopted threshold for systemic bacterial infection. Leukocyte cut-offs of < 4 × 10^9/L or > 12 × 10^9/L reflect standard SIRS limits. The 36 h look-back window was selected a priori to match routine postoperative testing intervals of approximately 48 h and biomarker kinetics, with PCT peaking at about 12–24 h and CRP at about 24–48 h, which supports temporal plausibility for fever preceding marker elevation. We compared the odds of being above threshold with and without a recent febrile episode using odds ratios and Fisher’s exact test p values. Because multiple draws per patient were analyzed the observations are not independent and p values may be optimistic, therefore we emphasize effect sizes and interpret p values cautiously. All analyses were considered exploratory.

### Data Documentation

Patient health status, including any complications and medication administration, was documented throughout the study period. All routine measurements were recorded within the MEDOCS system, a regional healthcare documentation and communication network.

### Ethical considerations

The study adhered to the ethical principles outlined in the Declaration of Helsinki and received approval from the local ethics committee.

## Results

Over an 11-month period, 147 patients were enrolled in the study. Out of these, 103 patients were ultimately included in the final analysis, while 44 were excluded. Specifically, five patients were excluded due to accidental premature patch removal, 13 patients were removed due to incorrect patch application or partial detachment, four patients withdrew from the study voluntarily, 19 patients were excluded because they were either discharged too early or transferred to units not participating in the study, one patient passed away, one was excluded due to a failure to be read in time, and one patient was removed due to a technical failure of the patch. Seven patients were managed on the ICU and 96 on the general ward. Given the very small number of ICU cases and identical measurement protocol, no ICU subgroup analysis was undertaken. Results are reported for the pooled cohort.

### Demographic data

The cohort included 103 patients with 54 men and 49 women. Age and BMI distributions are reported in Table 1. 33 patients, 17 men and 16 women, had documented fever and provide the surgery and medication details. In this febrile subgroup, surgery types clustered in hepato-pancreato-biliary procedures (13 of 33, 39.4%) and colorectal surgery (11 of 33, 33.3%), with smaller numbers for upper-gastrointestinal surgery (2 of 33, 6.1%), small bowel (1 of 33, 3.0%), urologic (1 of 33, 3.0%), and abdominal wall procedures (1 of 33, 3.0%). Antipyretic use occurred in all febrile patients (33 of 33, 100.0%) while NSAID exposure occurred in 4 of 33 patients (12.1%). Surgery and medication data were analysed only for the febrile subgroup and percentages for these items use subgroup denominators.

### Primary endpoint & clinical interventions related to fever detection

Among the 103 patients analyzed, 33 experienced at least one episode of fever during their hospital stay, as detected by either the wearable patch, the infrared thermometer, or both. The wearable patch identified fever in 31 out of these 33 patients, whereas the infrared thermometer detected fever in only 12 cases. Notably, in 21 patients, the infrared thermometer failed to detect fever entirely, while the wearable patch missed fever detected by infrared thermometry in only 2 cases.

Regarding clinical interventions triggered by fever detection, 19 of the 33 febrile patients required medical intervention during hospitalization, which included antibiotic therapy and surgical procedures. In 16 cases, antibiotic treatment was either initiated or adjusted, with the wearable patch detecting fever in 15 of these cases, whereas the infrared thermometer identified fever in only 7 cases. Additionally, in 5 patients, surgical intervention was deemed necessary due to their condition. The wearable patch successfully detected fever in all five cases, whereas the infrared thermometer identified fever in only two of these patients (Table 2).

Among patients with documented fever, 14 episodes did not lead to antibiotic modification or surgical intervention. Reasons were not standardized in routine charts. A review of the notes suggests common considerations such as short, self-limited spikes, attribution to clear non-infectious causes, and a lack of corroborating clinical or laboratory correlates. Because indications were not systematically recorded, these cases should be interpreted with caution and are acknowledged as a study limitation.

Among the 103 evaluable patients, no infection-related antibiotic adjustments or surgical interventions occurred in the absence of a documented fever episode (≥ 38.0 °C). All recorded infection-directed interventions were preceded by or coincident with fever documented by either the continuous patch or routine infrared charting.

### Results of the points-based system

The evaluation using the points-based system demonstrated a clear advantage for the wearable patch over the infrared thermometer in detecting clinically relevant fever events. The wearable patch achieved a total score of 880 points, while the infrared thermometer accumulated only 282 points. Breaking down the scoring components, the wearable patch detected 39 fever spikes lasting less than 60 min, contributing 39 points, and 77 fever episodes lasting 60 min or longer, resulting in 231 points. Additionally, fever detection by the patch was associated with 18 antibiotic interventions, leading to 360 points, and 5 cases requiring surgical intervention, adding 250 points to the total score. In contrast, the infrared thermometer recorded only 7 fever spikes shorter than 60 min (7 points) and 5 prolonged fever episodes (15 points). It detected fever in 8 cases that required antibiotic intervention, contributing 160 points, and in 2 cases where surgical intervention was necessary, adding 100 points to the total score (Table 3).

**Fig. 1:**
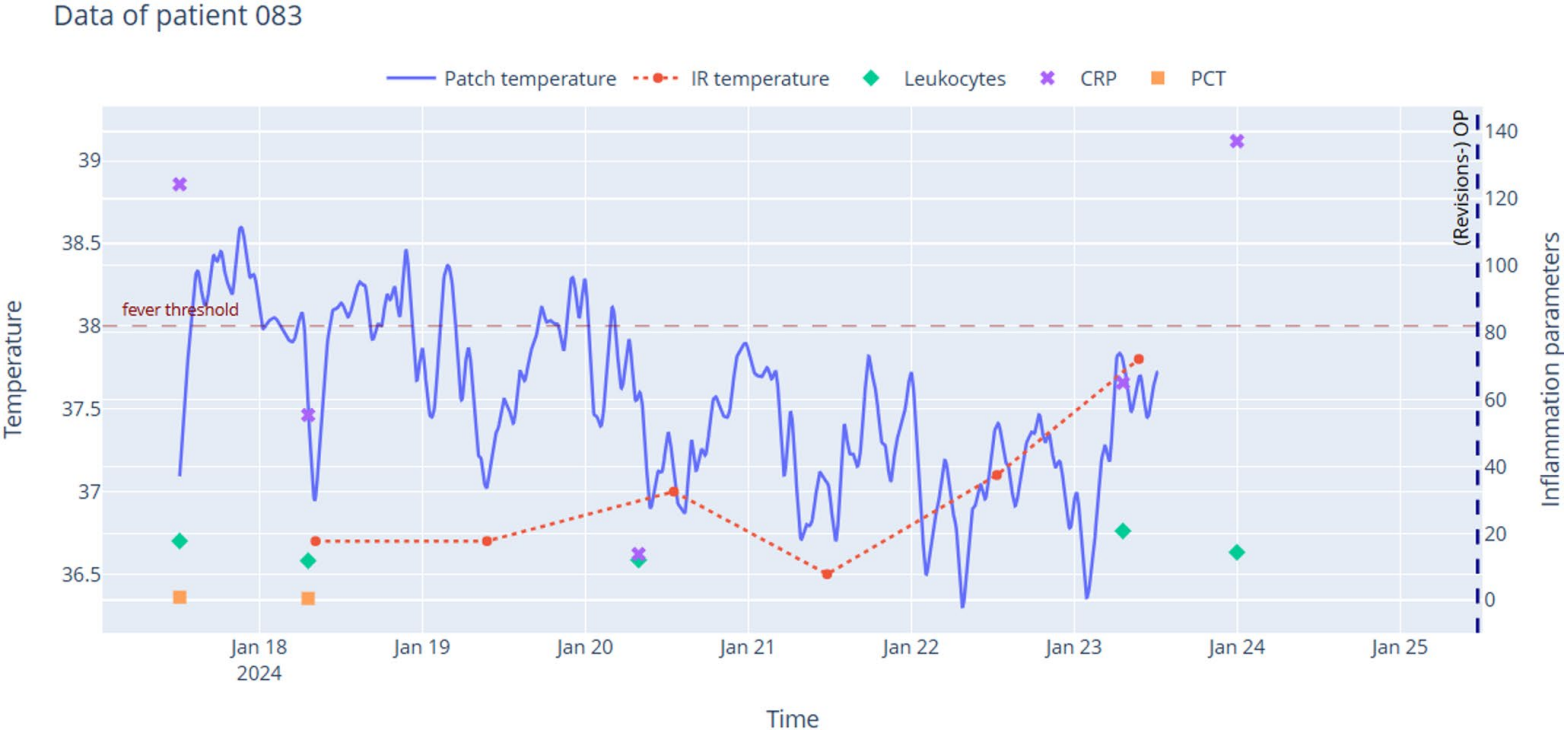
Temperature chart of patient #083. The wearable patch detected multiple prolonged fever episodes in a patient who ultimately required surgical intervention, whereas the infrared measurement did not register any fever episodes

**Fig. 2:**
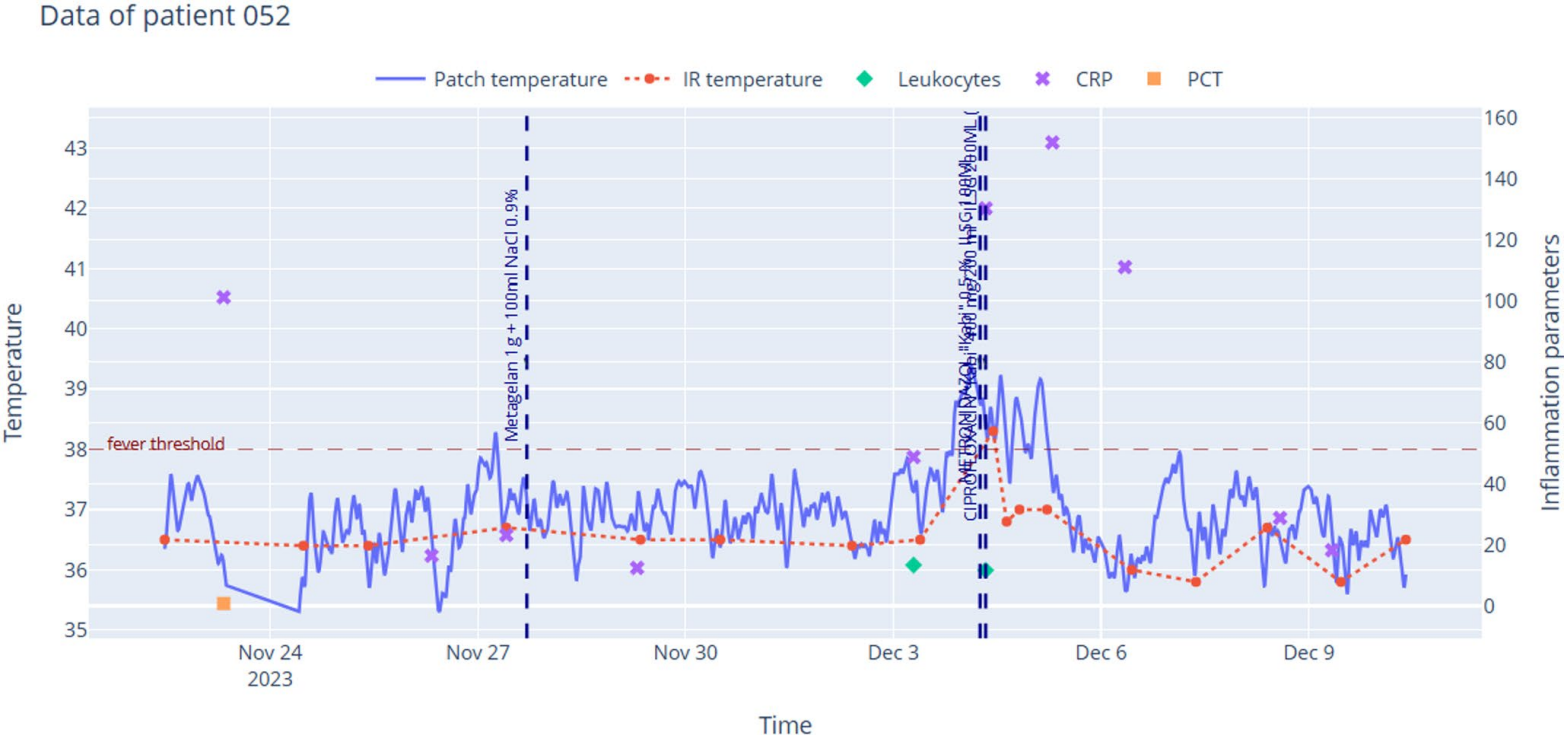
Temperature chart of patient #052. The infrared measurement detected fever events less frequently than the wearable patch. When fever was detected via infrared thermometry, it occurred with a 14-hour delay, after the necessary surgical intervention had already been performed

**Fig. 3:**
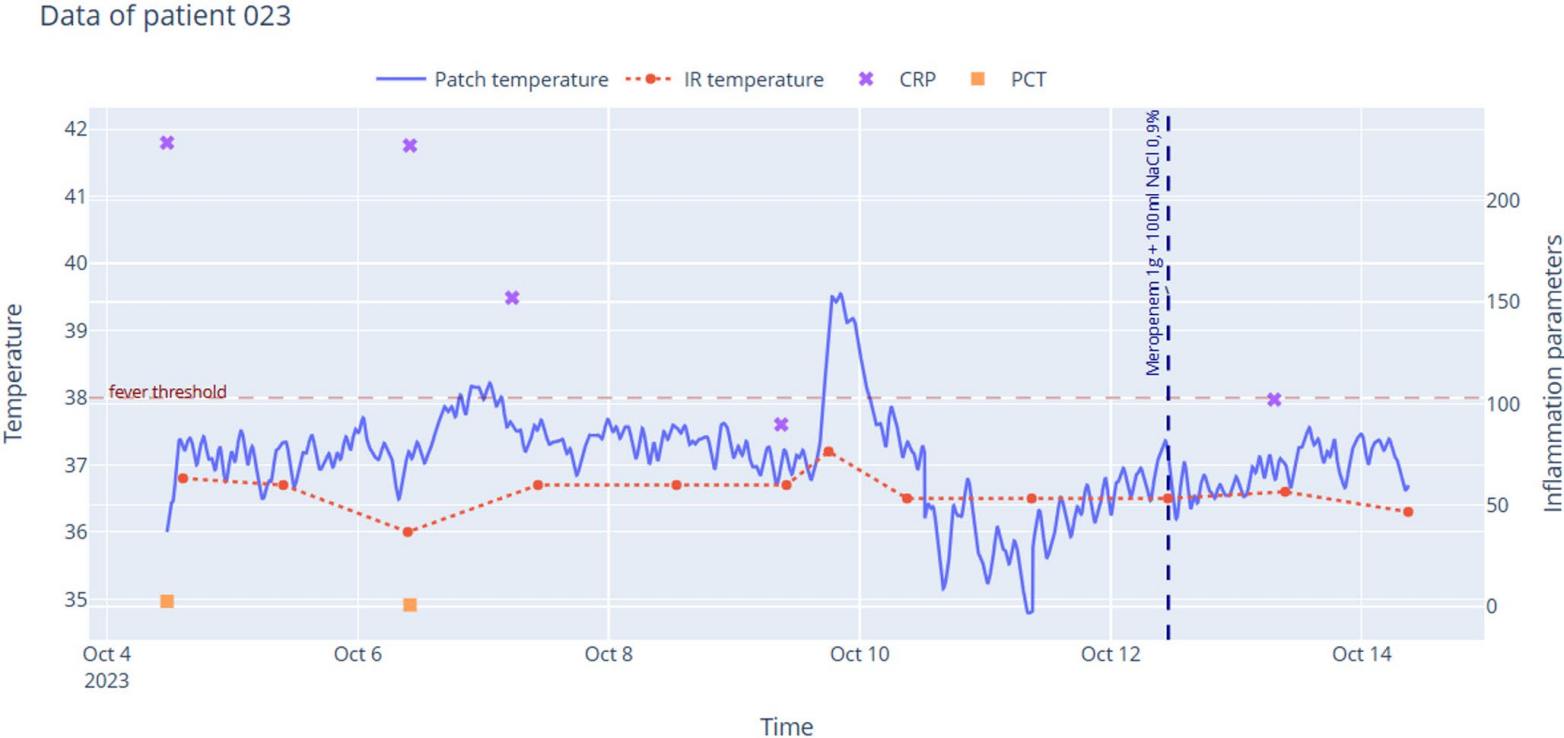
Body temperature and CRP levels of patient #023 over time. During the first days of hospitalization, CRP values exceeded 200 mg/L, while body temperature remained below the fever threshold. The wearable patch detected two fever episodes in a patient who later required antibiotic intervention, whereas the infrared measurement did not register any fever episodes

Using this evaluation system, continuous temperature monitoring via the wearable patch identified clinically relevant fever events ~ 3.12 times more frequently than single-point measurements using infrared thermometry. Additionally, infrared thermometry measurements often exhibited substantial discrepancies compared to patch-based readings (Figs. 1, 2 and 3). Even during fever events detected by the patch, infrared thermometry measurements frequently recorded significantly lower body temperatures. In some cases, infrared measurements closely aligned with patch readings when taken simultaneously; however, they failed to capture fever spikes or episodes that occurred between measurements, often missing such events completely.

### Secondary endpoint

**Fig. 4:**
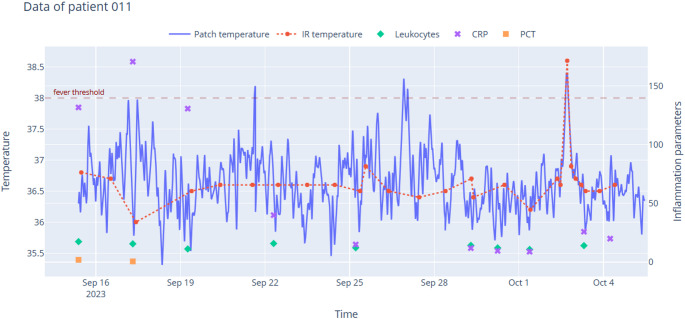
Body temperature and CRP levels of patient #011 over time. The initial temperature remained below the fever threshold, while CRP levels exceeded 100 mg/L. Later, CRP levels decreased below 50 mg/L, and multiple fever spikes were observed

Regarding the secondary endpoint, post-hoc exploratory analyses indicated a small and heterogeneous association between fever and inflammatory markers. CRP was more often above 100 mg/L within 36 h after a febrile episode (OR 3.54, Fisher *p* ≈ 0.001; medians 137.4 vs. 78.8 mg/L, Mann–Whitney *p* ≈ 0.007). PCT and leukocyte count showed limited or inconclusive discrimination in this cohort (PCT OR ≈ 0.53, *p* = 1.000; leukocytes OR ≈ 0.34, *p* ≈ 0.076).(Fig. 4)

### Inflammatory marker association

Across 33 patient datasets with continuous temperature monitoring, fever was detected in 31. In 22 of 33 datasets we identified 39 febrile episodes, defined as patch temperature ≥ 38.0 °C sustained for ≥ 60 min. 38 of these episodes had at least one CRP draw within the subsequent 36 h. After excluding laboratory tests obtained within the first 12 h after initiation of patch recording, we analyzed CRP (*n* = 146 draws), PCT (*n* = 22), and leukocytes (*n* = 62). Each laboratory draw was classified as fever-linked if a febrile episode had occurred within the prior 36 h, otherwise it was classified as non-fever-linked. For CRP with a threshold of > 100 mg/L, 38 of 146 draws were fever-linked and 26 of these 38 values, corresponding to 68.4%, exceeded the threshold; among the 108 non-fever-linked draws, 41 values, corresponding to 38.0%, exceeded the threshold. The resulting odds ratio was 3.54 with Fisher’s exact *p* ≈ 0.001, and the median CRP was higher when a recent fever was present at 137.4 mg/L versus 78.8 mg/L with a Mann-Whitney *p* ≈ 0.007. For PCT with a threshold of > 0.5 ng/mL, 5 of 22 draws were fever-linked and 4 of these, corresponding to 80.0%, exceeded the threshold; among the 17 non-fever-linked draws, 15 values, corresponding to 88.2%, exceeded the threshold. The odds ratio was approximately 0.53 with Fisher *p* = 1.000, and the medians were 0.6 versus 0.7 ng/mL with a Mann-Whitney *p* ≈ 0.433, indicating limited informativeness given the small sample. For leukocytes, defined as abnormal when < 4 × 10^9/L or > 12 × 10^9/L, 19 of 62 draws were fever-linked and 10 of these, corresponding to 52.6%, were abnormal; among the 43 non-fever-linked draws, 33 values, corresponding to 76.7%, were abnormal. The odds ratio was approximately 0.34 with Fisher *p* ≈ 0.076, and the medians were 12.0 versus 13.3 × 10^9/L with a Mann-Whitney *p* ≈ 0.129.

**Fig. 5:**
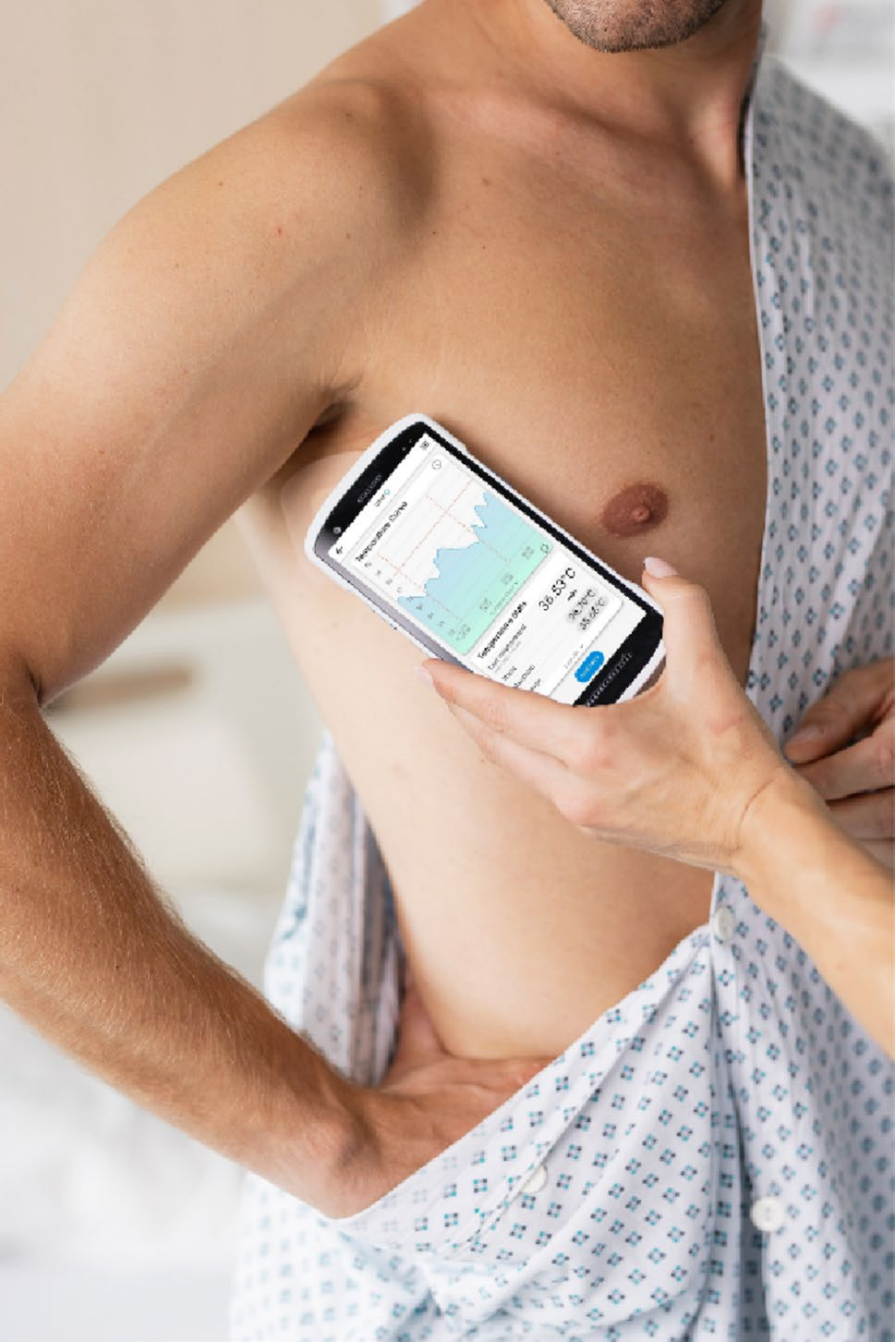
Reading of the patch using the Steadytemp® application

## Discussion

This study highlights the value of continuous temperature monitoring in postoperative care, emphasizing its effectiveness in detecting clinically relevant fever events. We could demonstrate that continuous monitoring using a wearable patch significantly outperforms intermittent infrared measurements in detecting fever. The wearable patch detected fever in 94% of patients with fever episodes (31 out of 33), while the infrared thermometer identified fever in only 36% (12 out of 33). Strikingly, the infrared thermometer failed to detect fever in 64% of the cases (21 patients). These findings suggest that the reliability of infrared thermometers in clinical practice should be critically reevaluated.

The principal advantage of the wearable patch lies in its continuous temporal resolution compared with single-point infrared measurements. A measurement site/device difference (axillary patch vs. temporal-artery infrared) may also contribute - the present design does not disentangle these components.

### Comparison with previous studies

Our results are consistent with findings from previous research on continuous temperature tracking. Flora et al. [14] examined high-frequency temperature monitoring in cancer patients and found that continuous measurements detected febrile adverse events several hours earlier than conventional monitoring. While our study focused on postoperative infections, both studies emphasize the superior sensitivity of continuous monitoring in detecting temperature deviations at an earlier stage.

Similarly, Jordan et al. [15] and Sampson et al. [16] explored the feasibility of wearable temperature sensors for continuous fever detection. Their findings support the idea that continuous monitoring enables earlier recognition of temperature fluctuations. Our study builds on these insights by specifically assessing the clinical relevance of early fever detection in a postoperative setting.

Wang et al. [17] analyzed the impact of temperature measurement timings on fever detection rates after gastrointestinal surgery. Their study found that the effectiveness of intermittent fever detection was highly dependent on the frequency and timing of measurements. Specifically, the fever detection rate varied from as low as 3.3% with a single daily measurement to 85% with 24 measurements per day, highlighting the high likelihood of missing fever episodes with standard intermittent approaches.

Our study supports these findings and demonstrates that continuous temperature monitoring offers a consistent and earlier fever detection. Implementing continuous temperature monitoring systems, could significantly enhance postoperative patient management.

### Insights on the primary endpoint

The primary endpoint - the ability to detect fever earlier - could not be evaluated using traditional statistical methods due to the frequent failure of the infrared thermometer to identify fever (Figs. 1, 2 and 3). However, the results obtained from the applied points-based system emphasize the clinical relevance of continuous temperature monitoring. Fever spikes detected by the patch were frequently associated with necessary medical interventions, such as initiating or adjusting antibiotic therapy (16 cases) and surgical interventions (5 cases). Notably, all five patients who required surgical intervention were flagged by the patch, whereas the infrared thermometer identified fever in only two of these cases. This discrepancy underscores the potential of continuous temperature monitoring in reducing delays in clinical decision-making and intervention.

### Method comparison using bland-altman analysis

**Fig. 6:**
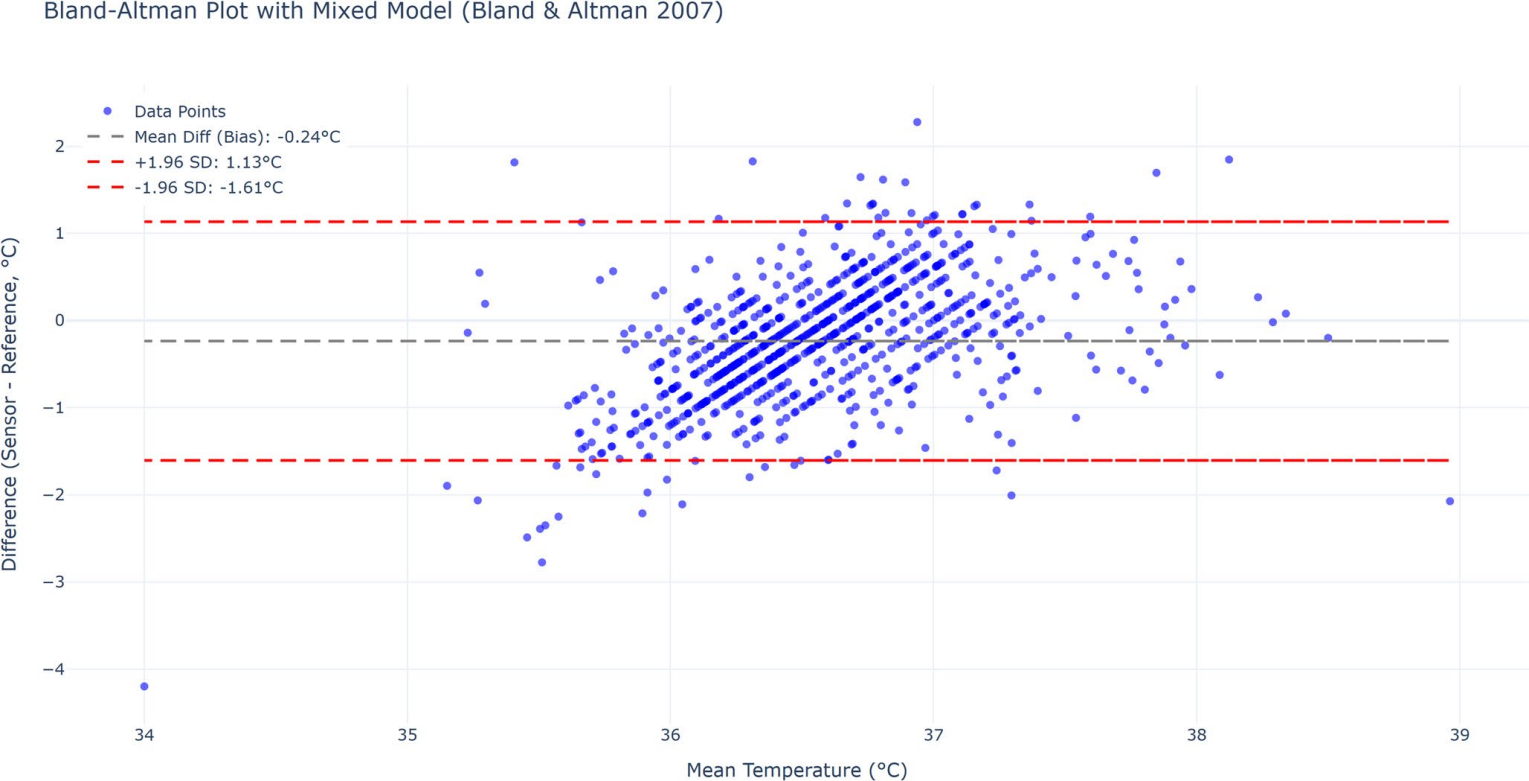
Bland-Altman plot showing agreement between SteadyTemp and reference thermometer for all time-matched paired measurements (*n* = 886). A mixed-effects model was used to account for repeated measures. The mean bias was − 0.237 °C, with 95% limits of agreement ranging from − 1.606 to 1.133 °C

To assess the agreement between the continuous monitoring system and the intermittent infrared method, a Bland-Altman analysis for repeated measurements was conducted using a mixed-effects model. This analysis revealed a small overall bias of − 0.237 °C, with relatively wide 95% limits of agreement (–1.606 to 1.133 °C) (Fig. 6).

**Fig. 7:**
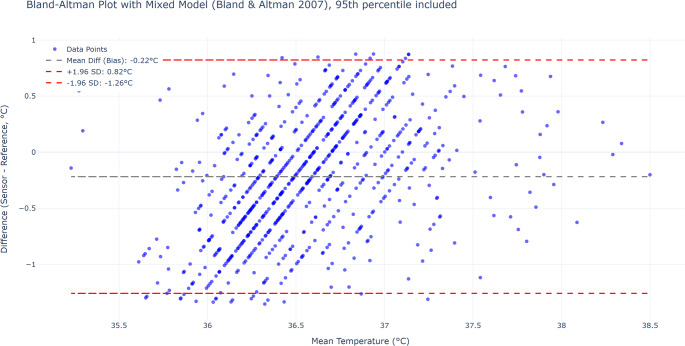
Bland-Altman plot after excluding the top and bottom 5% of measurement differences (*n* = 796). This approach aims to minimize the influence of short-term artifacts or extreme deviations.The adjusted mean bias and limits of agreement (LoA) were slightly improved (Bias: − 0.22 °C; LoA: − 1.26 to 0.82 °C)

**Fig. 8:**
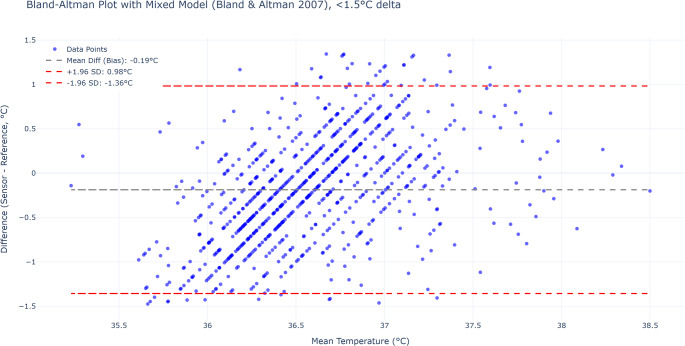
Bland-Altman plot after excluding the points where the absolute value of the measurement difference was < 1.5 °C. The adjusted mean bias was slightly improved, while the limits of agreement (LoA) were enhanced (Bias: − 0.19 °C; LoA: − 1.36 to 0.98 °C)

Several factors must be considered when interpreting these results. The Bland-Altman method compares paired measurements at individual time points and therefore does not account for the temporal dynamics captured by continuous monitoring. Transient physiological variations or external factors (e.g., patient hygiene, ambient temperature) may influence patch readings but are likely to be missed by intermittent spot measurements. Moreover, in some cases, patch readings consistently differed from the reference values in a patient-specific manner, which may reflect individual skin characteristics, sensor positioning, or physiological variability. To further explore the impact of such factors, sensitivity analyses were conducted excluding data points identified as potential artifacts based on predefined thresholds. These analyses resulted in slightly narrower limits of agreement (Figs. 7 and 8), suggesting that targeted data cleaning can enhance the robustness of method comparison in the context of continuous monitoring. Despite the methodological limitations inherent to Bland-Altman analysis in this context, the findings support the notion that continous temperature monitoring captures a broader spectrum of physiological temperature variation. Continuous measurements can reflect clinically relevant fluctuations - such as brief febrile episodes - that may be undetected by intermittent monitoring alone.

These observations highlight a fundamental limitation of single-point comparison methods like Bland-Altman plots when evaluating continuous monitoring systems: they fail to reflect the temporal resolution and additional clinical information provided by such technologies. Therefore, while statistical agreement is important, it should be interpreted alongside the added clinical value of continuous monitoring, particularly in detecting early or short-lived temperature elevations.

### Insights on the secondary endpoint

Regarding the secondary endpoint, post hoc exploratory analyses anchored on laboratory timestamps indicated a small and heterogeneous association between fever and inflammatory markers. The clearest signal was seen for CRP, whereas PCT and leukocyte count were less informative in this dataset. Figures 3 and 4 provide descriptive patient-anchored trajectories and were retained unchanged. The inferential results complement these figures. These findings are consistent with biomarker kinetics in which fever often precedes laboratory changes and they support the added value of continuous temperature monitoring for timelier detection while warranting cautious interpretation given the exploratory design and repeated measurements per patient. The apparent difference between Figs. 3 and 4 and the inferential results reflects distinct analytic frames.

### Infrared thermometers: a questionable standard

The inability of the infrared thermometer to detect fever in most cases brings its clinical reliability into question. Infrared thermometers are widely used due to their ease of use and perceived efficiency [18], but our findings clearly demonstrate their limitations. In patients with rising signs of infections, where early detection of fever is critical for timely interventions [14], relying on intermittent measurements may delay diagnosis and intervention. As such, the role of infrared thermometers in clinical practice should be reconsidered, particularly for vulnerable patient populations requiring close monitoring. Continuous monitoring technologies might offer a more robust and accurate alternative, ensuring that subtle temperature changes are not missed.

### Clinical relevance and practical considerations

Our results suggest that continuous temperature monitoring can improve the quality of care. Earlier fever detection has the potential to trigger timely diagnostic evaluations and therapeutic interventions, such as initiating antibiotics, which can reduce the risk of complications and shorten hospital stays. This approach could be particularly beneficial in intensive care or surgical units, where postoperative infections can rapidly escalate if not promptly addressed [19].

However, our study also revealed challenges regarding patch adherence and occasional premature removal. These challenges highlight the need for comprehensive staff training and communication with patients when introducing new monitoring technologies.

### Study limitations

While this study demonstrates the significant advantages of continuous temperature monitoring using a wearable patch, several limitations have to be acknowledged.

The study was conducted at a single center with a relatively small sample size of 103 patients included in the final analysis. Although the results are promising, they may not be generalizable to larger, more diverse patient populations or different clinical settings. Multi-Center studies with larger cohorts are needed to confirm the findings and assess the broader applicability of continuous temperature monitoring.

Some technical challenges were encountered, particularly related to patch adherence. In 13 cases, the patch was either incorrectly applied or partially detached. These issues underscore the importance of training clinical staff in the proper application of the patch and optimizing its design for better adhesion.

While the points-based evaluation system provides valuable insights, it may lack the statistical robustness of conventional analyses and could be considered subjective. The use of a heuristic, non-validated scoring scheme may limit generalizability and requires external validation.

**Table 1:** Demographic and clinical characteristics

Variable	Total (*n* = 103)	Men (*n* = 54)	Women (*n* = 49)
Age Distribution (%):			
20–29 years	3 (2.9%)	2 (3.7%)	1 (2.0%)
30–39 years	7 (6.8%)	2 (3.7%)	5 (10.2%)
40–49 years	11 (10.7%)	5 (9.3%)	6 (12.2%)
50–59 years	20 (19.4%)	8 (14.8%)	12 (24.5%)
60–69 years	29 (28.2%)	17 (31.5%)	12 (24.5%)
70–79 years	25 (24.3%)	16 (29.6%)	9 (18.4%)
80–89 years	8 (7.8%)	4 (7.4%)	4 (8.2%)
BMI Distribution (%):			
14–15.9.9	1 (1.0%)	0 (0%)	1 (2.0%)
16–17.9.9	2 (1.9%)	0 (0%)	2 (4.1%)
18–19.9.9	9 (8.7%)	2 (3.7%)	7 (14.3%)
20–21.9.9	21 (20.4%)	10 (18.5%)	11 (22.4%)
22–23.9.9	22 (21.4%)	11 (20.4%)	11 (22.4%)
24–25.9.9	20 (19.4%)	13 (24.1%)	7 (14.3%)
26–27.9.9	9 (8.7%)	6 (11.1%)	3 (6.1%)
28–29.9.9	8 (7.8%)	3 (5.6%)	5 (10.2%)
30–31.9.9	8 (7.8%)	6 (11.1%)	2 (4.1%)
32–32.9.9	3 (2.9%)	3 (5.6%)	0 (0%)
Febrile subgroup	Total (*n* = 33)	Men (*n* = 17)	Women (= 16)
Surgery categories			
Hepato-pancreato-biliary	13 (39.4%)	5 (29.4%)	8 (50.0%)
Colorectal	11 (33.3%)	6 (35.3%)	5 (31.2%)
Other	4 (12.1%)	2 (11.8%)	2 (12.5%)
Upper GI	2 (6.1%)	2 (11.8%)	0 (0.0%)
Abdominal wall	1 (3.0%)	1 (5.9%)	0 (0.0%)
Small bowel	1 (3.0%)	0 (0.0%)	1 (6.2%)
Urologic	1 (3.0%)	1 (5.9%)	0 (0.0%)
Medication exposure			
Antipyretics	33 (100.0%)	17 (100.0%)	16 (100.0%)
NSAIDs	4 (12.1%)	3 (17.6%)	1 (6.2%)

Factors such as used medications, patient movement, and variations in ambient conditions may have influenced temperature readings and contribute to variability in measurements [20, 21]. Furthermore intermittent infrared thermometry obtained in routine care is susceptible to occasional operator-dependent misapplication (e.g., suboptimal site/scan technique or skin conditions) and timing effects, potentially introducing measurement error and misclassification of fever status.

**Table 2:** Fever Detection and Clinical Interventions

Category	Wearable Patch	Infrared Thermometer
Patients with fever (n = 33)	31/33 (93.9%)	12/33 (36.4%)
Missed fever cases	2	21
Antibiotic interventions (n = 16)	15/16 (93.8%)	7/16 (43.8%)
Surgical interventions (n = 5)	5/5 (100%)	2/5 (40%)

Figures 3 and 4 are descriptive visualizations and were retained unchanged for transparency. The inferential, laboratory-anchored analysis was performed post hoc and should be interpreted cautiously.

**Table 3:** Results of the Points-Based Evaluation System

Category	Wearable Patch	Infrared Thermometer
Total Score	880 points	282 points
Fever spikes (<60 min)	39 × 1 = 39	7 × 1 = 7
Fever episodes (>60 min)	77 × 3 = 231	5 × 3 = 15
Antibiotic interventions	18 × 20 = 360	8 × 20 = 160
Surgical interventions	5 × 50 = 250	2 × 50 = 100

Indications for initiating or withholding infection-directed treatment were not systematically documented in routine charts. Undocumented fever or non-febrile decisions may therefore not have been captured, although our chart review did not identify such cases.

These limitations highlight important considerations for the interpretation and broader implementation of the study findings. Despite these challenges, the results provide a strong basis for further research into continuous temperature monitoring technologies. Future studies should address these limitations by incorporating larger sample sizes and multi-center designs. Optimizing device design and improving integration into clinical workflows will be critical for realizing the full potential of continuous temperature monitoring in clinical practice. Additionally, continuous monitoring could potentially be expanded to track other vital parameters, such as heart rate and respiratory rate, further enhancing its clinical utility. The integration of machine learning-based predictive models to further refine fever detection scoring, ensuring even greater clinical applicability could also be explored.

In summary, our study highlights the clear advantages of continuous temperature monitoring using a wearable patch over intermittent infrared measurements. Continuous monitoring enables earlier and more reliable fever detection, facilitates timely interventions, and compensates for the limitations of routine blood tests in detecting early infection signs. Integrating continuous temperature monitoring into clinical practice offers a promising approach to improving patient safety, particularly in settings where early infection detection is critical.

## Data Availability

No datasets were generated or analysed during the current study.
